# Supplementary material for: Improving access to treatment for alcohol dependence in primary care: A qualitative investigation of factors that facilitate and impede treatment access and completion
Source: PLoS One. 2023 Oct 19;18(10):e0292220. doi: 10.1371/journal.pone.0292220 (PMC10586622; doi:10.1371/journal.pone.0292220)
Supplement: S1 File — (PDF) [file pone.0292220.s002.pdf]

Interview topic guide for service users

IRAS ID: 313497

Introduction

- On self
  - Research assistant at Liverpool John Moores University (LJMU), School of Psychology since February
  - Working with university staff of the psychology department and primary care staff such as GPs in Liverpool on this project
  - Interviewing adults who have experienced alcohol dependence in Liverpool
- Aims and purpose of the study
  - Explore how access to treatment for people with alcohol dependence and treatment itself can be improved in Liverpool
  - Hear your personal experience of support/treatment you may have received or, if you haven't, what you think of accessing and using support or treatment
- Confidentiality
  - Your interview and anything you speak about will be confidential: It will not be shared with anyone outside of the study team of LJMU, unless you tell me something that suggests that you might seriously harm yourself or others
- Rights
  - Can stop the interview at any point or take a break
  - Can skip questions if you do not want to answer
- Recording
  - Ok to record the interview?
- Any questions before we start?

Questions

1. About you:
   a. How old are you?/What is your age range? (younger than 20, 20s/30s/40s/50s/60s/older than 70?)

b. What is your gender?
c. Do you currently work? If so, what do you do?
d. Which part of Liverpool do you live in?
e. Which ethnic group do you identify with?
    - Asian or Asian British
    - Black, Black British, Caribbean or African
    - Mixed or multiple ethnic groups
    - White
    - Other ethnic group

2. Alcohol dependence: We would like to know a little more about your experiences related to your use of alcohol. How long have you been dependent? When did you get a diagnosis of alcohol dependence? Where did this happen?
3. Treatment access: We would like to know about your experiences and thoughts about alcohol treatment and getting access to it
    a. Have you ever been treated in primary care for reasons linked to your use of alcohol?
        - Access: If so, what treatment did you receive? How many times? What made you start treatment? How did you get access to treatment? What was your experience of this process?
        - Access: If not, what are the reasons you have not received any treatment? What are your expectations of treatment?
4. Barriers and facilitators
    a. If you have accessed treatment before, what was treatment like for you? What challenges did you encounter (if any)?
    b. What went well?
5. Types of treatment
    a. Have you received any other (medical/clinical/social) support for reasons linked to your use of alcohol?

- If so, what? What made you use this type of support? How did you get access to it? What was your experience of this process and the support itself?
- If no support, what are the reasons you have not received any? If you would like support, what type?

b. Have you received any support/care from specialist alcohol services?

- If so, what? What was your experience of it?

c. Have you ever been offered anti-craving medication?

- If so, what medication? Did you use it? How did this go?
- If not, what are your thoughts about anti-craving medication?

6. Improvement of treatment access + treatment
    - How would you change treatment for people with alcohol dependence to make it better?
    - What would help people to access treatment?
7. Would you like to add anything?
