# Supplementary material for: Improving access to treatment for alcohol dependence in primary care: A qualitative investigation of factors that facilitate and impede treatment access and completion
Source: PLoS One. 2023 Oct 19;18(10):e0292220. doi: 10.1371/journal.pone.0292220 (PMC10586622; doi:10.1371/journal.pone.0292220)
Supplement: S2 File — (PDF) [file pone.0292220.s003.pdf]

Interview topic guide for healthcare professionals

IRAS ID: 313497

Introduction

- On self
    - Research assistant at Liverpool John Moores University (LJMU), School of Psychology since February
    - Working with university staff of the psychology department and primary care staff such as GPs in Liverpool on this project
    - Interviewing healthcare professionals involved in service provision to adults with alcohol dependence in Liverpool
- Aims and purpose of the study
    - Explore how access to treatment for people with alcohol dependence and treatment itself can be improved in Liverpool
    - Hear your experience of providing support or treatment to people with alcohol dependence
- Confidentiality
    - Your interview and anything you speak about will be confidential: It will not be shared with anyone outside of the study team of LJMU, unless you tell me something that suggests that you might seriously harm yourself or others
- Rights
    - Can stop the interview at any point or take a break
    - Can skip questions if you do not want to answer
- Recording
    - Ok to record the interview?
- Any questions before we start?

Questions:

1. About you:

a. How old are you?/What is your age range? (younger than 20, 20s/30s/40s/50s/60s/older than 70?)
b. What is your gender?
c. What is your job?
d. Which part of Liverpool do you live in?
e. Which ethnic group do you identify with?
    - Asian or Asian British
    - Black, Black British, Caribbean or African
    - Mixed or multiple ethnic groups
    - White
    - Other ethnic group

2. Treatment access: We would like to know your experiences and thoughts about providing services or treatment to people with alcohol dependence and treatment access for this group
    a. How have you been involved in facilitating access to treatment for people with alcohol dependence?
        - What is your experience of this process?
            1. What challenges do you encounter (if any)?
            2. What is working well?
3. Barriers and facilitators
    a. What support or treatment do you provide/have you provided to people with alcohol dependence?
        - What challenges do you encounter (if any) in supporting patients to engage with treatment? What challenges in supporting patients to complete treatment?
        - Any challenges in supporting patients to engage with or complete treatment that are specific to certain patient groups?
        - What is going/did go well?
4. Types of treatment

a. Have you provided/referred patients to Brief Interventions?
    - If not, what are the reasons you have not done so?
    - If so, what was your experience of it?

b. Have you prescribed/informed patients of/supported the use of anti-craving medication?
    - If so, what medication? What were patients' experiences/responses to this?
    - If not, what are the reasons for this? What are your thoughts about anti-craving medication?

5. Improvement of treatment access + treatment
    - How would you change treatment for people with alcohol dependence to make it better?
    - What would help people to access treatment?
6. Would you like to add anything?
