# Supplementary material for: Improving access to treatment for alcohol dependence in primary care: A qualitative investigation of factors that facilitate and impede treatment access and completion
Source: PLoS One. 2023 Oct 19;18(10):e0292220. doi: 10.1371/journal.pone.0292220 (PMC10586622; doi:10.1371/journal.pone.0292220)
Supplement: Attachment — Submitted filename: Response to Reviewers_R2.docx [file pone.0292220.s005.docx]

8^th^ September 2023

Dear Dr White,

Thank you for your email dated 4^th^ August regarding our manuscript *“Improving access to treatment for alcohol dependence in primary care: A qualitative investigation of factors that facilitate and impede treatment access and completion”*.

We are most grateful to you and the reviewers for their comments on the manuscript. We have made changes to the existing version of the manuscript to take account of the reviewers’ comments, and we hope that this is now suitable for publication in PLOS ONE.

Regarding Reviewer 2’s comment:

*“Results. With regard to the details of patients (Table 1), it would have been interesting to know at what stage of AD they were, since how long that had been diagnosed, what treatments they had used.”*

Unfortunately we do not have this level of detail for all participants as some participants were less forthcoming with this information, and it we did not systematically collect this data as it was not a key aim of the study. As per your suggestion we have noted this as a limitation in lines 417-419 of the manuscript.

We hope that this is now suitable for publication in PLOS ONE.

Yours Sincerely,

Cathy

Dr Cathy Montgomery

Reader in Psychopharmacology

Liverpool John Moores University

Liverpool, UK.
